# Everolimus pharmacokinetics and its exposure–toxicity relationship in patients with thyroid cancer

**DOI:** 10.1007/s00280-016-3050-6

**Published:** 2016-05-11

**Authors:** D. de Wit, T. C. Schneider, D. J. A. R. Moes, C. F. M. Roozen, J. den Hartigh, H. Gelderblom, H. J. Guchelaar, J. J. van der Hoeven, T. P. Links, E. Kapiteijn, N. P. van Erp

**Affiliations:** Department of Clinical Pharmacy and Toxicology, Leiden University Medical Center, Leiden, The Netherlands; Department of Medical Oncology, Leiden University Medical Center, Leiden, The Netherlands; Department of Endocrinology, University Medical Center Groningen, Groningen, The Netherlands; Department of Clinical Pharmacy, Radboud University Medical Center, Postbus 9101, 6500 HB Nijmegen, The Netherlands

**Keywords:** Everolimus, Exposure–toxicity, Population pharmacokinetics, Pharmacogenetics, Individualized dosing

## Abstract

**Background:**

Everolimus is a mTOR inhibitor used for the treatment of different solid malignancies. Many patients treated with the registered fixed 10 mg dose once daily are in need of dose interruptions, reductions or treatment discontinuation due to severe adverse events. This study determined the correlation between systemic everolimus exposure and toxicity. Additionally, the effect of different covariates on everolimus pharmacokinetics (PK) was explored.

**Methods:**

Forty-two patients with advanced thyroid carcinoma were treated with 10 mg everolimus once daily. Serial pharmacokinetic sampling was performed on days 1 and 15. Subsequently, a population PK model was developed using NONMEM to estimate individual PK values used for analysis of an exposure–toxicity relationship. Furthermore, this model was used to investigate the influence of patient characteristics and genetic polymorphisms in genes coding for enzymes relevant in everolimus PK.

**Results:**

Patients who required a dose reduction (*n* = 18) due to toxicity at any time during treatment had significant higher everolimus exposures [mean AUC_0–24_ (SD) 600 (274) vs. 395 (129) µg h/L, *P* = 0.008] than patients without a dose reduction (*n* = 22). A significant association between everolimus exposure and stomatitis was found in the four-level ordered logistic regression analysis (*P* = 0.047). The presence of at least one TTT haplotype in the *ABCB1* gene was associated with a 21 % decrease in everolimus exposure.

**Conclusion:**

The current study showed that dose reductions and everolimus-induced stomatitis were strongly associated with systemic everolimus drug exposure in patients with cancer. Our findings confirm observations from another study in patients with cancer and show us that everolimus is a good candidate for individualized dosing in patients with cancer.

**ClinicalTrial.gov number:**

NCT01118065.

**Electronic supplementary material:**

The online version of this article (doi:10.1007/s00280-016-3050-6) contains supplementary material, which is available to authorized users.

## Introduction

Everolimus is an orally administered rapamycin derivative inhibiting the mammalian target of rapamycin (mTOR) [1]. This is a key signaling molecule in the phosphatidylinositol 3-kinase (PI3K)/Akt pathway which is involved in the regulation of growth, proliferation, metabolism, survival and angiogenesis of cells and often dysregulated in cancer [1]. Currently, everolimus is registered for the treatment of advanced hormone receptor positive (HR^+^), human epidermal growth factor-2 negative (HER2^−^) breast cancer in postmenopausal women in combination with exemestane, for metastatic renal cell carcinoma (mRCC), and for irresectable or metastatic pancreatic neuroendocrine tumors (pNET) and subependymal giant cell astrocytoma (SEGA) [2–4].

Despite its proven efficacy, everolimus is also associated with a number of serious side effects. Most common toxicities associated with everolimus therapy include stomatitis, rash, fatigue, diarrhea, infections, nausea, loss of appetite, hematologic toxicities, dyspnea, noninfectious pneumonitis and metabolic abnormalities such as hypercholesterolemia and hyperglycemia [5]. While it is reported that the majority of these adverse events are manageable and of mild-to-moderate severity, many patients are in need of dose interruptions, reductions or treatment discontinuation due to toxicity [6]. Indeed, in the pivotal breast cancer, mRCC and pNET phase III trials, 10–35 % of the patients discontinued everolimus treatment due to adverse events [2–4]. In addition, ~62 % of the patients needed dose interruptions or reductions compared to 12–29 % in the placebo arms [2, 4].

The large number of dose reductions and treatment discontinuation make toxicity currently one of the main challenges in the optimal use of everolimus for the treatment of cancer. In oncology, everolimus is registered as a fixed oral dose of 10 mg once daily. However, in transplantation medicine therapeutic drug monitoring (TDM) with individualized dosing is routinely applied due to everolimus’ narrow therapeutic window and high inter-patient variability in pharmacokinetics (PK) [7]. In transplantation medicine, everolimus is used as an immunosuppressant to prevent rejections. Dose individualization is not only applied to prevent toxicity, but also to optimize treatment efficacy. In oncology, the same high inter-patient variability in PK is seen (AUC; 45 CV%, *C*_trough_; 60 CV%) [8]. This substantial variability, in combination with the fixed 10 mg dosing, results in large differences in everolimus exposure between patients. This could result in either supra-therapeutic drug exposure with an increased incidence of toxicity, but also in subtherapeutic drug exposure leading to decreased anticancer effects.

The primary objective of this study was to assess the correlation between everolimus exposure and toxicity in patients with advanced thyroid cancer. Additionally, we explored the influence of different covariates on everolimus PK, including genetic polymorphisms in genes encoding enzymes involved in the absorption and metabolism of everolimus.

## Materials and methods

### Patients

Forty-two patients were enrolled in this phase II study investigating the efficacy and PK of everolimus for the treatment of progressive or recurrent, unresectable or metastatic thyroid cancer. The efficacy data of this study will be reported separately. Participating medical centers were the Leiden University Medical Center and the University Medical Center Groningen. Patients were treated continuously with everolimus at an once-daily oral dose of 10 mg until tumor progression, unacceptable toxicity, death or discontinuation from the study for other reasons. Toxicities were assessed at baseline, days 1, 14 and 28 of therapy and monthly thereafter according to the National Cancer Institute (NCI) Common Terminology Criteria for Adverse Events (CTC-AE) version 4.0. Dose adjustments were permitted for adverse events suspected to be related to everolimus. The first dose reduction was to 5 mg once daily. If another dose reduction was needed, everolimus was dosed as 5 mg every other day. The study was approved by the institutional ethics committees (Leiden University Medical Center and University Medical Center Groningen, the Netherlands), and all patients gave written informed consent before entering the study.

### Pharmacokinetic sample collection and analysis

For everolimus PK assessment, whole blood samples were obtained at days 1 and 15 of therapy. Samples were collected into EDTA tubes at predose and 1, 2 and 3 h after everolimus intake (sparse schedule). More extensive PK sampling at 4, 5, 6, 7 and 8 h after everolimus intake was optional for patients (extensive schedule). Samples were stored at −20 °C until the day of analysis.

Everolimus concentrations in whole blood were measured using a validated ultra performance liquid chromatography-tandem mass spectrometric (UPLC-MS/MS) assay. Validation of the assay was performed according to the EMA guidelines of bioanalytical method development [9]. The calibration line was linear over the range from 2 to 160 µg/L, and the lower limit of quantification (LLOQ) was 0.6 µg/L. Assay performance was in agreement with guidelines for bioanalytical method development and validation.

### Pharmacogenetic analysis

#### Single-nucleotide polymorphisms and haplotype selection

Everolimus is metabolized by the cytochrome P450 (CYP) enzymes CYP3A4, CYP3A5 and CYP2C8 and is also a substrate for the efflux pump P-glycoprotein (P-gp) encoded by the *ABCB1* gene [10]. The nuclear pregnane X receptor (PXR; NR1I2) regulates the expression of CYP3A4 and could therefore also influence everolimus PK [11]. Eleven single-nucleotide polymorphisms (SNPs) in these genes were selected based upon a candidate gene approach (Supplementary Data S1, online). For the *ABCB1* and *CYP2C8* gene, selected SNPs were used for haplotype analysis performed in gPLINK (Supplementary Data S2, online). Haplotypes were set at a certainty >0.97. For the *ABCB1* and *CYP2C8* genes, only haplotypes and no individual SNPs were tested.

#### Genotyping assays

Germline DNA was isolated from 400 µl EDTA blood using MagNa Pure Compact (Roche, Almere, the Netherlands). DNA concentrations were thereafter measured using Nanodrop (Isogen, De Meern, the Netherlands). Genotyping was performed using predesigned genotyping assays (Supplementary Data S1, online). Samples were analyzed on a Viia7 real-time PCR system according to the manufacturers’ instruction (Life Technologies, Bleiswijk, the Netherlands). Call rates of all assays were >98 %. As a quality control, at least 5 % of the samples were genotyped in duplicate. No inconsistencies were observed. Minor allele frequencies (MAF) of all 11 SNPs were calculated and compared with reported MAF for European Populations (HAPMAP). No significant deviations were observed, and derived allele frequencies were all in Hardy–Weinberg equilibrium (*P* ≥ 0.05) (Supplementary Data S1, online).

### Pharmacokinetic modeling

#### Base model

Thirty patients completed the extensive PK sampling and ten patients the sparse PK sampling schedule. After PK sampling, nonlinear mixed-effects modeling (NONMEM) was used to describe the population PK of everolimus. Subsequently, the developed population PK model was used to estimate individual everolimus exposure both in terms of AUC_0–24_ by using clearance and with use of the model predicted *C*_trough_ levels. NONMEM version 7.2 (Icon Development Solutions, Ellicott City, MD, USA) was used with Piranã (version 2.9.0) as the modeling environment. Statistical software package R (version 2.15.1) was used for handling of data and plot generation. We also used NONMEM to explore the influence of different covariates on everolimus PK.

A first-order conditional estimation method with interaction (FOCE-I) was used to fit models throughout the building process. One- and two-compartment models with first-order elimination were explored. It was also assessed whether there was a change in clearance from day 1 to day 15 of treatment. Model selection was based on goodness of fit and statistical significance. An adjusted model was chosen over the original model if the drop in the objective function value (OFVs) was >3.84 [*P* < 0.05 with one degree of freedom (*df*), assuming *χ*^2^-distribution].

Since the bioavailability (*F*) of everolimus is unknown, *F* was fixed at 1 and PK parameter estimates reported are proportional to *F* except *K*_a_. In addition, both clearance (Cl/*F*) and the volume of distribution (*V*_d_/*F*) were allometrically scaled [12].

#### Covariate analysis

After the base model was determined, covariates were tested to explore the influence of bilirubin, aspartate aminotransferase (ASAT), alanine aminotransferase (ALAT), creatinine, body surface area (BSA) and hematocrit on Cl/*F*. Individual effect sizes were estimated with the formula ${\text{Cl}}/F_{{{\text{typical}}\,{\text{value}}}} = \theta_{1} \times ({\text{COV}}/{\text{COV}}_{\text{median}} )^{{\theta_{2} }}$, whereby *θ*_1_ is the population estimate for Cl/*F*, COV the tested covariate and *θ*_2_ the covariant effect size estimate.

The influence of SNPs and haplotypes was all tested as a covariate on Cl/F, except for *ABCB1* haplotypes which were tested for an effect on *F* as this is physiologically more plausible. Effect sizes were estimated with the formula Cl/*F*_typical value_ (or *F*) = $\theta_{1} \times \theta_{2}^{{{\text{pg}}1}} \times \theta_{3}^{{{\text{pg}}2}} ,$ whereby *θ*_1_ is the population Cl/*F* or *F* estimate in wild-type patients, *θ*_2_ the covariate effect size of the heterozygote mutation status and *θ*_3_ the effect size of the homozygote mutation status. The heterozygote (pg1) and homozygote (pg2) mutation status was scored as 1 if present or 0 if not present. If the genotype frequency was <0.1, homozygote mutant and heterozygote mutant genotypes were combined (Supplementary Data S1 and S2, online).

All covariates were first tested for statistical significance with univariate forward inclusion into the base model (drop in OFV >3.84, *df* = 1, *P* < 0.05). After inclusion of significant covariates in the intermediate model, a stepwise backward elimination procedure was performed. Covariates were remained in the final model if the threshold for statistical significance of backward elimination was reached (increase in OFV >6.64, *df* = 1, *P* < 0.01).

#### Evaluation of model fit

Next to goodness-of-fit plots, a visual predictive check (VPC) was used to assess the performance of the final model by comparing the 10th and 90th percentiles of the simulated concentrations with those of the observed concentrations. In addition, a bootstrap analysis was performed to evaluate the precision of parameter estimation. Shrinkage in inter-individual variability and residual errors were automatically calculated by NONMEM.

### Assessment of systemic exposure toxicity relationship

#### Selection of toxicities

In this study, all experienced toxicities were scored according to CTC-AE version 4.0. However, due to the number of patients included, only a limited number of toxicities were selected to be tested for an association with everolimus exposure in order to prevent false-positive findings.

We choose dose reductions as the first outcome of toxicity as this is the sum of all different toxicities experienced by patients and these are also the toxicities that lead to clinical action by the treating physician. In addition, we selected stomatitis and pneumonitis as toxicity outcomes. The rationale for selection of these toxicities was based on their prevalence and the fact that these toxicities are (1) objectively measurable, (2) clinically relevant and (3) untreatable and therefore leading to dose reductions or discontinuation of therapy. Toxicities were scored as the highest grade experienced until dose reduction and if no reduction occurred until the end of study.

### Statistical analysis

The difference in day 15 steady-state everolimus exposure (AUC_0–24_ and C_trough_) between patients with and without dose reductions was tested with an unpaired *t* test. The relationships between day 15 everolimus exposure (AUC_0–24_ and *C*_trough_) and stomatitis and pneumonitis were evaluated using a four-level ordered logistic regression in SPSS version 20.0 (IBM).

## Results

### Patient characteristics

Forty-two adult patients with thyroid carcinoma, 22 men and 20 women were included in the phase II trial that investigated everolimus for the treatment of thyroid cancer. Of these patients, 28 (66.7 %) had differentiated, 7 (16.7 %) had undifferentiated (anaplastic) and 7 (16.7 %) had medullary advanced thyroid carcinoma. Two patients were excluded for PK analysis; in one patient, no PK samples were collected, and in the other patient, no measurable everolimus levels could be detected. Patient baseline characteristics are shown in Table 1.

**Table 1:** Patient baseline characteristics

Characteristics	
*N*	40
Age (years)	63 (40–80)
Gender (*n*)	
Male	21 (52.5 %)
Female	19 (47.5 %)
Length (cm)	173 (154–189)
Weight (kg)	75 (45–105)
Hematology	
WBC (×10^9^/L)	7.1 (3.6–25)
ANC (×10^9^/L)	4.8 (2.7–13.0)
Platelets (×10^9^/L)	254 (147–995)
Hemoglobin (mmol/L)	7.5 (5.3–10.7)
Hematocrit	0.39 (0.29–0.50)
Chemistry	
AST (U/L)	22 (12–61)
ALT (U/L)	22 (7–19)
Creatinine (µmol/L)	66 (42–205)
Total bilirubin (µmol/L)	9 (4–16)
Tumor type (*n*)	
Differentiated	26 (65 %)
Undifferentiated	7 (17.5 %)
Medullary	7 (17.5 %)

Data are presented as median (range) unless stated otherwise

*ALT* alanine aminotransferase, *ANC* absolute neutrophil count, *AST* aspartate aminotransferase, *WBC* white blood count

### Pharmacokinetics

A total of 669 samples from 40 patients were used to build the population PK model. The pharmacokinetic data for everolimus were best described by a two-compartmental model with first-order absorption and first-order elimination from the central compartment (Supplementary Data S3, online). No difference in clearance over time between day 1 and day 15 of treatment was found.

Forward inclusion of BSA, creatinine, ASAT, ALAT, bilirubin and hematocrit did not improve the PK model, and no association between these covariates and clearance was found. With forward inclusion of the *ABCB1* TTT and CCG haplotype, the base model significantly improved (ΔOFV = −7.2 and −6.4, respectively, *P* < 0.05). The other SNPs and the *CYP2C8* haplotype did not improve the model. With multivariate backward elimination, only the presence of at least one *ABCB1* TTT haplotype remained significant (ΔOFV = 9.6, *P* < 0.01). A 21 % decrease in *F* was observed in the presence of at least one *ABCB1* TTT haplotype. Inclusion of this covariate in the final PK model reduced the inter-patient variability in Cl/*F* from 38.1 to 35.1 CV%. Parameter estimates of the base and final model are shown in Table 2.

**Table 2:** Summary of model parameter estimates

Parameter	Base model			Final model			Bootstrap runs	
	Estimate	RSE (%)	Shrinkage (%)	Estimate	RSE (%)	Shrinkage (%)	Median value	95 % CI
Cl/*F* (L/h)	20.3	7.0		17.4	8.4		18.0	15.5–20.8
*F*	1	–		1	–		1	–
*V*_1_/*F* (L)	29.1	18.5		25.2	17.8		25.7	18.1–40.4
*k*_a_ (h^−1^)	0.643	5.3		0.647	6.2		0.653	0.583–0.740
*Q* (L/h)	60	4.7		51.1	7.3		52.1	45.5–59.1
*V*_2_ (L)	475	5.4		400	–		400	–
*θ*_TTT_ on *F*	NA	NA		0.792	6.5 %		0.81	0.71–0.90
Inter-individual variability								
Cl/*F* (CV%)	38.1 %	34.4	10	35.1 %	30.5	11	35.0 %	22.1–49.1 %
*V*_1_/*F* (CV%)	87.3 %	35.7	27	86.4 %	35.3	27	90.5 %	53.7–138.9 %
Inter-occasion variability								
*F* (CV%)	20.7 %	37.7	9	19.2 %	38.1	12	19.4 %	12.9–30.5 %
Residual variability								
* σ* (proportional error)	27.2 %	20.7	7	27.3 %	20.8	7	27.9 %	22.6–32.9 %

**Fig. 1:**
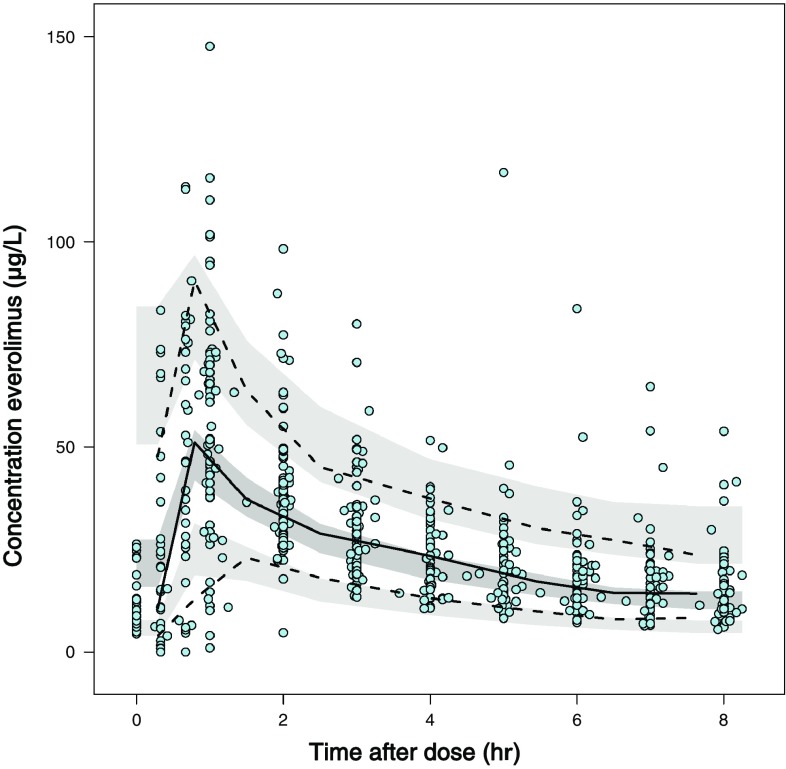
Visual predictive check (VPC) of final everolimus PK model

Evaluation of the final model was, next to inspection of the goodness-of-fit plots, done with VPC and a bootstrap procedure. Results of the VPC show that predicted and observed concentration intervals are almost identical, indicating accuracy and good predictive performance of the final model (Fig. 1). There is a small tendency for a difference between predicted and observed concentrations in the absorption part of the curve due to limited number of samples during this phase. Since we mainly used the model to estimate individual values for Cl/*F*, the modest under-prediction of the absorption did not affect our analysis. The successful bootstrap procedure with 1000 runs is shown in Table 2. The median values for PK parameters found were within 10 % of those estimated with the final model, indicating that the model is precise and reliable in its parameter estimation.

### Exposure–toxicity relationship

The relationships between everolimus exposure and dose reductions as well as stomatitis and pneumonitis were examined. In total, 45 % of the patients had their everolimus 10 mg dose reduced to a lower dose due to toxicity (Table 3). In general, toxicity developed within 3 months after the start of everolimus therapy. Toxicities leading to dose reduction included stomatitis, pneumonitis, fatigue, loss of appetite, diarrhea, liver and kidney toxicity and edema. Considering stomatitis, 42.5 % of the patients experienced any grade stomatitis and 7.5 % experienced grade 3 stomatitis. In addition, 10 % of the patients had a noninfectious pneumonitis.

**Table 3:** Dose reductions and toxicity incidence

Dose reductions	
No	22 (55 %)
Yes	18 (45 %)
Stomatitis	
None	23 (57.5 %)
Grade 1	12 (30 %)
Grade 2	2 (5 %)
Grade 3	3 (7.5 %)
Pneumonitis	
None	36 (90 %)
Grade 1	2 (5 %)
Grade 2	1 (2.5 %)
Grade 3	1 (2.5 %)
Reason for reduction	
Stomatitis	4 (22.2 %)
Pneumonitis	4 (22.2 %)
Fatigue	5 (27.8 %)
Loss of appetite	1 (5.6 %)
Diarrhea	1 (5.6 %)
Liver toxicity	1 (5.6 %)
Kidney toxicity	1 (5.6 %)
Edema	1 (5.6 %)

**Fig. 2:**
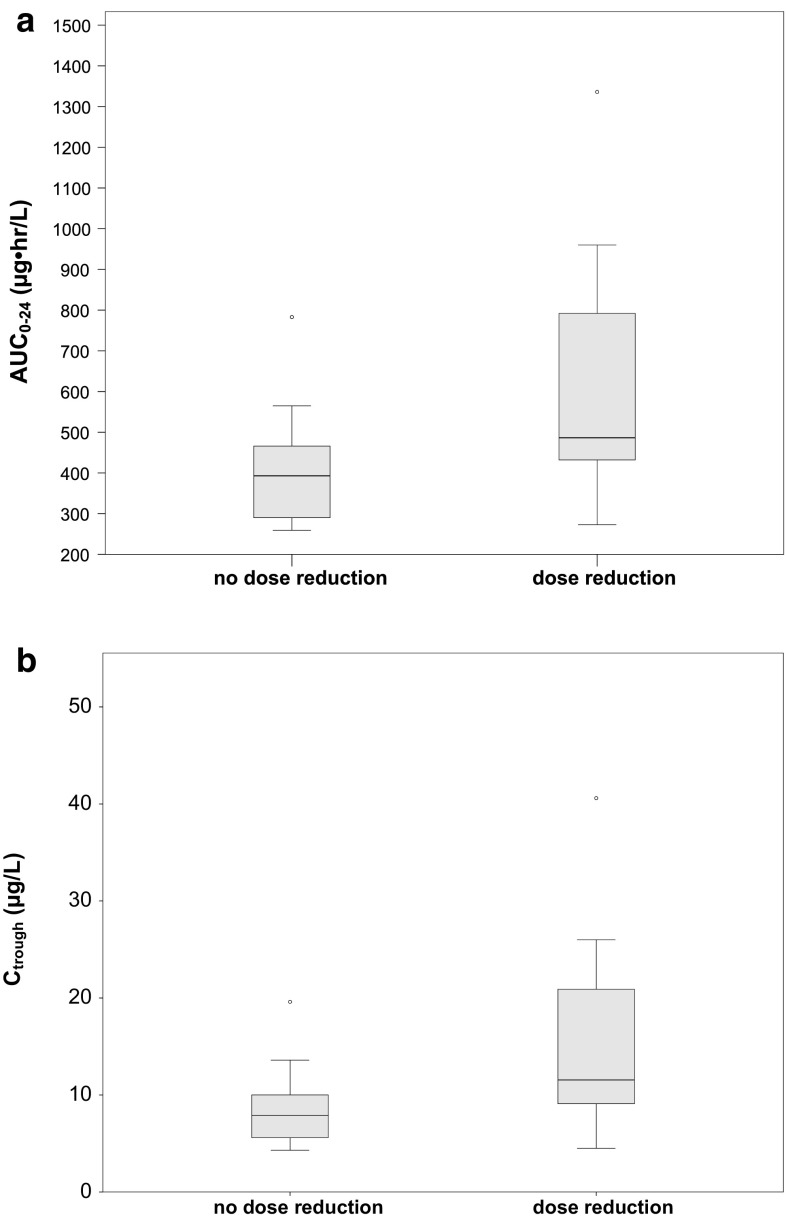
*Boxplot* of everolimus exposure in patient with and without dose reduction. **a** AUC_0–24_ and **b** *C*_trough_

**Fig. 3:**
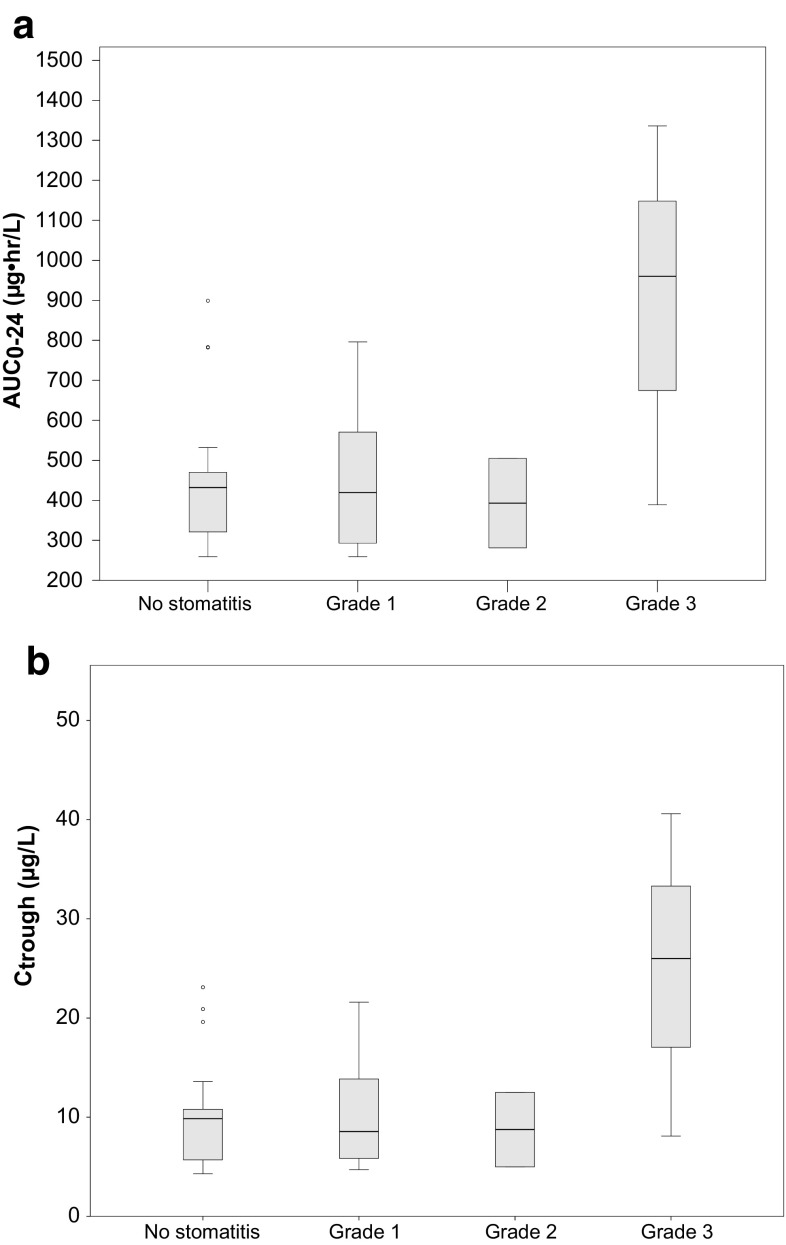
*Boxplot* of severity of stomatitis versus everolimus exposure. **a** AUC_0–24_ and **b** *C*_trough_

Figure 2 shows boxplots of everolimus AUC and C_trough_ in patients with and without dose reduction. Mean AUC_0–24_(SD) and *C*_trough_ were 600 (274) and 395 (129) µg h/L and 14.9 (9.0) and 8.4 (3.8) µg/L for patients with and without dose reductions, respectively. The exposure to everolimus was significantly different between the two groups (mean difference in AUC −204 µg h/L (95 % CI −340 to −69 µg h/L, *P* = 0.008 and mean difference in *C*_trough_ −6.5 µg/L (95 % CI −11.2 to −1.8 *P* = 0.009). Figure 3 shows boxplots of AUCs and *C*_trough_ in patients experiencing different grades of stomatitis. A positive association between everolimus exposure and stomatitis was identified (*P* = 0.047). The odd ratio for stomatitis was 1.16 (95 % CI; 1.06–1.26) for every 50 µg h/L increase in AUC_0–24_. Patients with grade 3 stomatitis had an everolimus exposure that was two times that of patients with ≤2 stomatitis (AUC_0–24_ 896 vs. 456 µg h/L, *P* > 0.05 and *C*_trough_ 24.9 vs. 10.3 µg/L, *P* > 0.05). No association of everolimus exposure with pneumonitis was found.

## Discussion

This study was primarily performed to assess the correlation between everolimus exposure and toxicity. Results show that patients who had their everolimus dose reduced due to toxicity had significantly higher drug exposures than patients without the need for dose reductions. Moreover, everolimus exposure was associated with the probability for stomatitis and patients with grade 3 stomatitis had an everolimus exposure two times that of patients with ≤2 stomatitis. Additionally, we found that the presence of at least one TTT allele in the *ABCB1* gene was associated with lower everolimus exposure due to decreased absorption.

The present findings of a clear relationship between everolimus exposure and dose reductions due to toxicity and stomatitis are in line with results from other studies in patients with cancer treated with everolimus. Previously, it has been shown that a twofold increase in everolimus exposure increased the risk of ≥grade 3 pulmonary events, ≥grade 3 stomatitis and ≥grade 3 metabolic events with 1.9-fold, 1.5-fold and 1.3-fold, respectively, in patients with advanced solid tumors [13]. The present analysis could not confirm the earlier identified association of everolimus exposure with pneumonitis, but this may be due to the limited number of patients with pneumonitis in our study cohort.

The fixed 10-mg dosing regimen of everolimus is based on its safety profile together with its pharmacodynamic effects on the mTOR-dependent pathway in tumor and skin biopsies [14, 15]. These studies suggested a dose of ≥5 or ≥10 mg daily, based on complete inhibition of serine/threonine kinase p70S6 kinase (S6K1) or phosphorylated eIF-4G (peIF-4G) which are both downstream targets of mTOR. Since inhibition of peIF-4G was only complete at the 10-mg dose level, it was advised to use the 10-mg once-daily schedule for future clinical studies. The clinical relevance of this difference in inhibition of eIF-4G is, however, unknown and should be further investigated as higher dosing introduces also more toxicity.

The present study underscores the high inter-patient variability in everolimus PK which is in line with previous observations [8]. This is also analog to the variability in PK seen for other oral targeted therapies for the treatment of cancer such as tyrosine kinase inhibitors (TKIs). For TKIs, the evidence for relationships between systemic drug exposure and efficacy or toxicity endpoints is growing [16, 17]. The currently available data suggest that an individualized dosing approach seems justified in certain circumstances and different studies support the feasibility of an individualized dosing approach for TKIs [18, 19].

In the exploration of covariates of influence on everolimus PK, the presence of at least one TTT haplotype was responsible for a decrease in everolimus exposure due to decreased absorption. Previously, the TTT haplotype has been demonstrated to be associated with enhanced function of the P-glycoprotein transporter and indeed reduced exposure or efficacy of treatment [20–22]. However, decreased function of the transporter and thus increased exposure have also been reported, as well as studies that could not show an effect [7, 23, 24]. The association we found should be regarded as preliminary and needs further validation. If this association is confirmed, it might be argued whether a decrease in exposure of 21 % can be considered as clinically relevant when taking into account the inter-patient variability in everolimus PK.

To the best of our knowledge, we are the first to describe the population PK of everolimus 10 mg once daily in patient with cancer. Previously, population PK models have been described, but only within the field of transplantation medicine where everolimus is used in a much lower dose. Taking this and differences in modeling into account, pharmacokinetic parameter estimates were in agreement with those previously found [7].

Everolimus exposures were assessed at day 15 of therapy and not necessarily at the time when adverse events occurred. This may be considered as a limitation, and future studies should preferably measure everolimus exposure at the time that toxicity occurs. However, the variability in everolimus PK within a patient (intra-patient) is reported to be much smaller than the variability between patients [25, 26]. In addition, we observed a constant clearance of everolimus over time. While treated at the same dose (10 mg once daily), this restricts the probability for large differences between the exposures that we have measured and the actual exposures that would have been measured at the moment that toxicity occurred. In addition, the study that previously described a correlation between everolimus exposure and toxicity found similar results with the use of *C*_trough_ at the time of toxicity or when *C*_trough_ averaged over a given time period was used [13].

The present results both underscore the correlation between everolimus exposure and dose reductions due to toxicity as well as the high inter-patient variability in everolimus PK. These observations should be taken into account in the use of everolimus for the treatment of solid tumors. Preventing high drug exposures by dose individualization may have the potential to reduce the side effects of everolimus therapy while remaining its efficacy. However, prospective validation within oncology patients in necessary. Moreover, it has been shown that high early everolimus exposure (*C*_trough_ >14.1 µg/L) is associated with longer progression-free survival (PFS) and overall survival (OS) (13.3 and 26.2 vs. 3.9 and 9.9 months for PFS and OS, respectively) in patients with mRCC [27]. Hence, an individualized dosing approach may also be of value for some patients with treatment inefficacy due to subtherapeutic exposures. On the other hand, in this present analysis there were also patients in need of dose reductions in whom the exposure to everolimus was not elevated. This finding suggests that a subpopulation may not benefit from dose individualization but maybe more from treatment switch if available. In summary, future studies are required to define the therapeutic window of everolimus for the treatment of different malignancies and these studies should aim to optimize both treatment toxicity and efficacy outcomes possibly by using everolimus in a more individualized way.

## Conclusion

In conclusion, this study shows a clear association between everolimus exposure and dose reductions due to toxicity as well as stomatitis in patients with cancer using a newly developed population PK model. Our findings confirm observations from another study in patients with cancer and show us that everolimus is a good candidate for individualized dosing in patients with cancer.

## Electronic supplementary material

Below is the link to the electronic supplementary material.

Supplementary material 1 (DOCX 17 kb)

Supplementary material 2 (DOCX 14 kb)

Supplementary data 3. Schematic presentation of PK model (TIFF 502 kb)

Supplementary data 4. Goodness-of-fit (GOF) plot of individual predicted vs. observed everolimus concentrations (PDF 31 kb)
