# Supplementary material for: Everolimus pharmacokinetics and its exposure–toxicity relationship in patients with thyroid cancer
Source: Cancer Chemother Pharmacol. 2016 May 11;78:63–71. doi: 10.1007/s00280-016-3050-6 (PMC4921118; doi:10.1007/s00280-016-3050-6)
Supplement: Supplementary file 1 — Supplementary material 1 (DOCX 17 kb) [file 280_2016_3050_MOESM1_ESM.docx]

| **Supplementary data 1.** Selected polymorphisms in genes involved in the absorption and metabolism of everolimus | | | | | | | | | |
| --- | --- | --- | --- | --- | --- | --- | --- | --- | --- |
| **Gene** | **rs number** | **Polymorphism** | **Genotype** | **Frequency**  **N (%)** | | **Observed Minor Allele Frequency (%)** | **HWE (*p-*value)** | **Assay ID** | **Covariate testing** |
| ABCB1 | rs1128503 | 1236C>T | CC (wt)  TC  TT | 12  25  4 | (29.2)  (61.0)  (9.8) | T = 40.2% | 0.09 | C___7586662_10 | in haploblock |
| ABCB1 | rs2032582 | 2677G>T/A | GG (wt)  GT  TT | 12  23  5 | (30.0)  (57.5)  (12.5) | T = 41.3% | 0.24 | C_11711720C_30 | in haploblock |
| ABCB1 | rs1045642 | 3435T>C | TT (wt)  TC  CC | 10  24  7 | (24.4)  (58.5)  (17.1) | C = 46.3% | 0.26 | C___7586657_20 | in haploblock |
| NR1I2 | rs2276707 | 8055C>T | CC (wt)  CT  TT | 30  8  3 | (73.2)  (19.5)  (7.3) | T = 17.1% | 0.05 | C__15882324_10 | CC vs. CT+TT |
| NR1I2 | rs6785049 | 7635A>G | AA (wt)  AG  GG | 15  19  7 | (36.6)  (46.3)  (17.1) | G = 40.2% | 0.82 | C__29280426_10 | AA vs. AG vs. GG |
| CYP3A5 | rs776746 | 6986A>G | GG/*3*3(wt)  AG/*1*3  AA/*1*1 | 32  7  2 | (78.0)  (17.1)  (4.9) | A = 13.4% | 0.09 | C__26201809_30 | GG vs. AG+AA |
| CYP3A4 | rs2246709 | 16090A>G | AA (wt)  AG  GG | 19  15  6 | (47.5)  (37.5)  (15.0) | G = 33.8% | 0.31 | C___1845287_10 | AA vs. AG vs. GG |
| CYP2C8 | rs7909236 | -271G>T | GG (wt)  GT  TT | 21  19  1 | (51.2)  (46.4)  (2.4) | T = 25.6% | 0.17 | custom designed * | GG vs. GT+TT |
| CYP2C8 | rs10509681 | 47603213T>C | TT (wt)  CT | 36  5 | (87.8)  (12.2) | C = 6.1% | 0.68 | C__25625782_20 | in haploblock |
| CYP2C8 | rs11572080 | 47631494C>T | CC(wt)  TC | 36  5 | (87.8)  (12.2) | T = 6.1% | 0.68 | C__25625794_10 | in haploblock |
| CYP3A4 | rs35599367 | 522-191C>T | CC (wt)  CT | 39  2 | (95.1)  (4.9) | T = 4.9% | 0.87 | C_59013445_10 | not tested due to too low frequency |
| * custom designed assay: PCR primers, forward: 5’-GTATTGGATTGGAGCCCAGGTATTT-3’, reverse: 5’-TGTTTCTCCATCATCACAGCACAT-3’; probes, VIC: AAGTCCCTGGTTGTTCCA, FAM: TCCCTGGTTTTTCCA | | | | | | | | | |
