# Supplementary material for: Everolimus pharmacokinetics and its exposure–toxicity relationship in patients with thyroid cancer
Source: Cancer Chemother Pharmacol. 2016 May 11;78:63–71. doi: 10.1007/s00280-016-3050-6 (PMC4921118; doi:10.1007/s00280-016-3050-6)
Supplement: Supplementary file 2 — Supplementary material 2 (DOCX 14 kb) [file 280_2016_3050_MOESM2_ESM.docx]

| **Supplementary data 2**. Selected haploblocks in genes involved in the absorption and metabolism of everolimus | | | | | | | |
| --- | --- | --- | --- | --- | --- | --- | --- |
| **Gene** | **rs number** | **Polymorphism** | **Genotype** | **Frequency**  **N (%)** | | **Observed Minor Allele Frequency (%)** | **Covariate testing** |
| ABCB1 haploblock | rs1128503  rs2032582  rs1045642 | 1236C>T  2677G>T/A  3435T>C | other-other  TTT-other  TTT-TTT | 23  14  4 | (56.1)  (34.1)  (9.8) | TTT= 26.8% | other-other vs. TTT-other + TTT-TTT |
|  |  |  | other-other  CTG-other  CTG-CTG | 30  10  1 | (73.2)  (24.4)  2.4) | CTG = 14.6% | other-other vs. CTG-other + CTG-CTG |
|  |  |  | other-other  CCG-other  CCG-CCG | 10  26  5 | (24.4)  (63.4)  (12.2) | CCG = 43.9 | other-other vs. CCG-other vs. CCG-CCG |
| CYP2C8 haploblock | rs10509681  rs11572080 | 47603213T>C  47631494C>T | CT-CT  TC-CT | 36  5 | (87.8)  (12.2) | TC = 6.1% | CT-CT vs. TC-CT |
