# Supplementary figures and images for: Everolimus pharmacokinetics and its exposure–toxicity relationship in patients with thyroid cancer
Source: Cancer Chemother Pharmacol. 2016 May 11;78:63–71. doi: 10.1007/s00280-016-3050-6 (PMC4921118; doi:10.1007/s00280-016-3050-6)

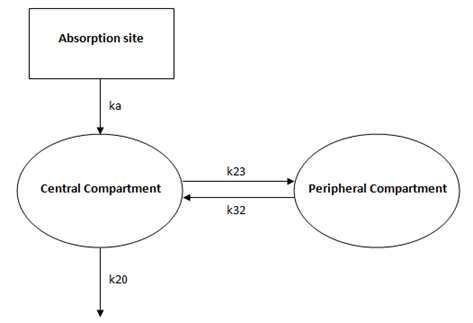

Supplement: Supplementary file 3 — Schematic presentation of PK model (TIFF 502 kb) [file 280_2016_3050_MOESM3_ESM.tif]

A: ADVAN5 COV\_GENETICA\_opF\_ABCB1\_CCG\_TTT\_BACKWARD DELETION

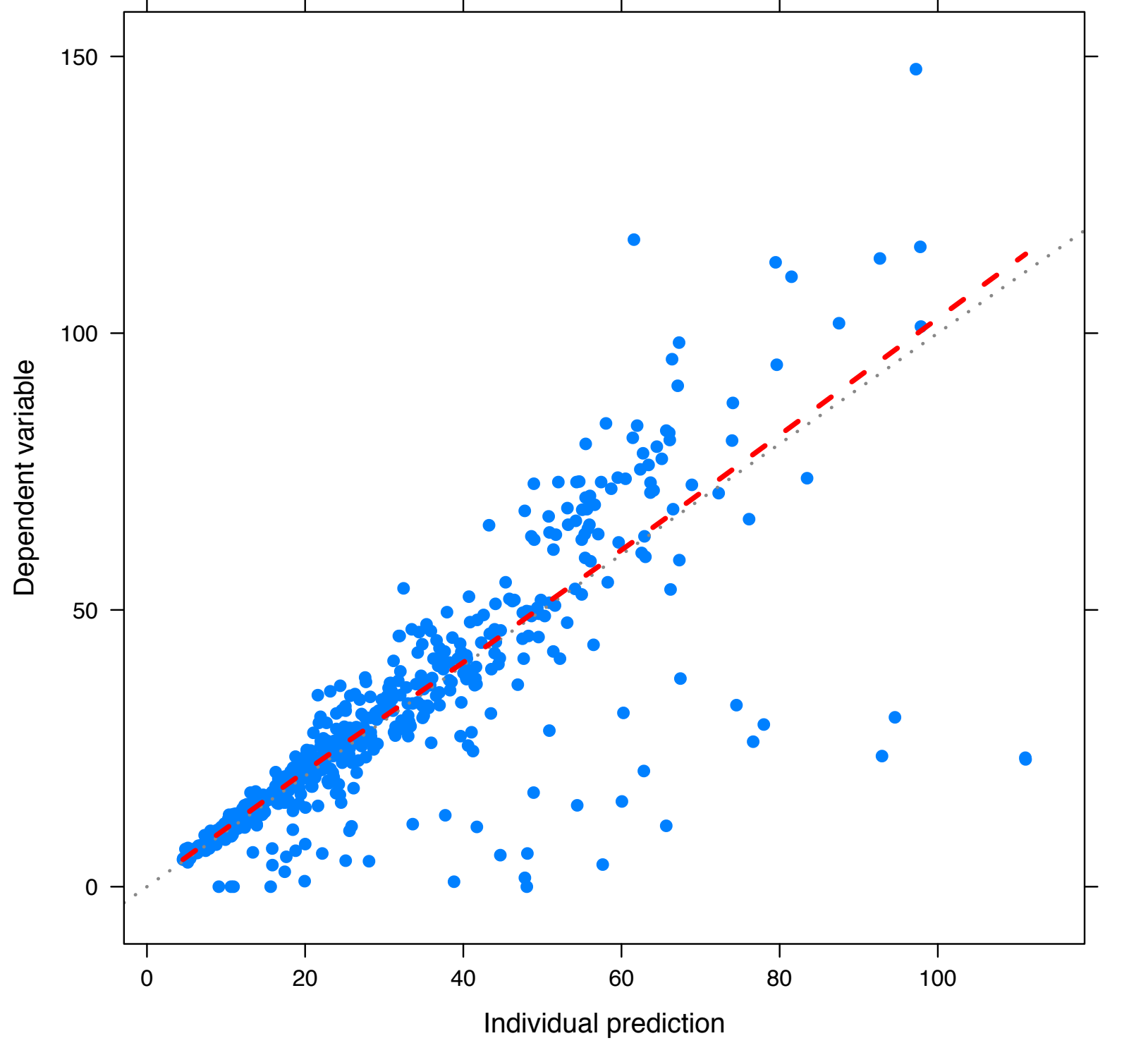

Supplement: Supplementary file 4 — Goodness-of-fit (GOF) plot of individual predicted vs. observed everolimus concentrations (PDF 31 kb) [file 280_2016_3050_MOESM4_ESM.pdf]
